# MTGO: PPI Network Analysis Via Topological and Functional Module Identification

**DOI:** 10.1038/s41598-018-23672-0

**Published:** 2018-04-03

**Authors:** Danila Vella, Simone Marini, Francesca Vitali, Dario Di Silvestre, Giancarlo Mauri, Riccardo Bellazzi

**Affiliations:** 1 Istituti Clinici Scientifici Maugeri, Pavia, Italy; 2 Department of Informatics, Systems and Communication, University of Milano-Bicocca, Milan, Italy; 3 Department of Computational Medicine and Bioinformatics, University of Michigan, Ann Arbor, MI USA; 4 Department of Electrical, Computer and Biomedical Engineering, University of Pavia, Pavia, Italy; 5 Centre for Health Technologies, University of Pavia, Pavia, Italy; 6 Center for Biomedical Informatics and Biostatistics, The University of Arizona Health Sciences, Tucson, AZ USA; 7 BIO5 Institute Center for Biomedical Informatics and Biostatistics, The University of Arizona Health Sciences, Tucson, AZ USA; 8 Department of Medicine, The University of Arizona Health Sciences, Tucson, AZ USA; 9 Institute of Biomedical Technologies National Research Council, Segrate, Italy

## Abstract

Protein-protein interaction (PPI) networks are viable tools to understand cell functions, disease machinery, and drug design/repositioning. Interpreting a PPI, however, it is a particularly challenging task because of network complexity. Several algorithms have been proposed for an automatic PPI interpretation, at first by solely considering the network topology, and later by integrating Gene Ontology (GO) terms as node similarity attributes. Here we present MTGO - Module detection via Topological information and GO knowledge, a novel functional module identification approach. MTGO let emerge the bimolecular machinery underpinning PPI networks by leveraging on both biological knowledge and topological properties. In particular, it directly exploits GO terms during the module assembling process, and labels each module with its best fit GO term, easing its functional interpretation. MTGO shows largely better results than other state of the art algorithms (including recent GO-based ones) when searching for small or sparse functional modules, while providing comparable or better results all other cases. MTGO correctly identifies molecular complexes and literature-consistent processes in an experimentally derived PPI network of Myocardial infarction. A software version of MTGO is available freely for non-commercial purposes at https://gitlab.com/d1vella/MTGO.

## Introduction

**Figure 1:**
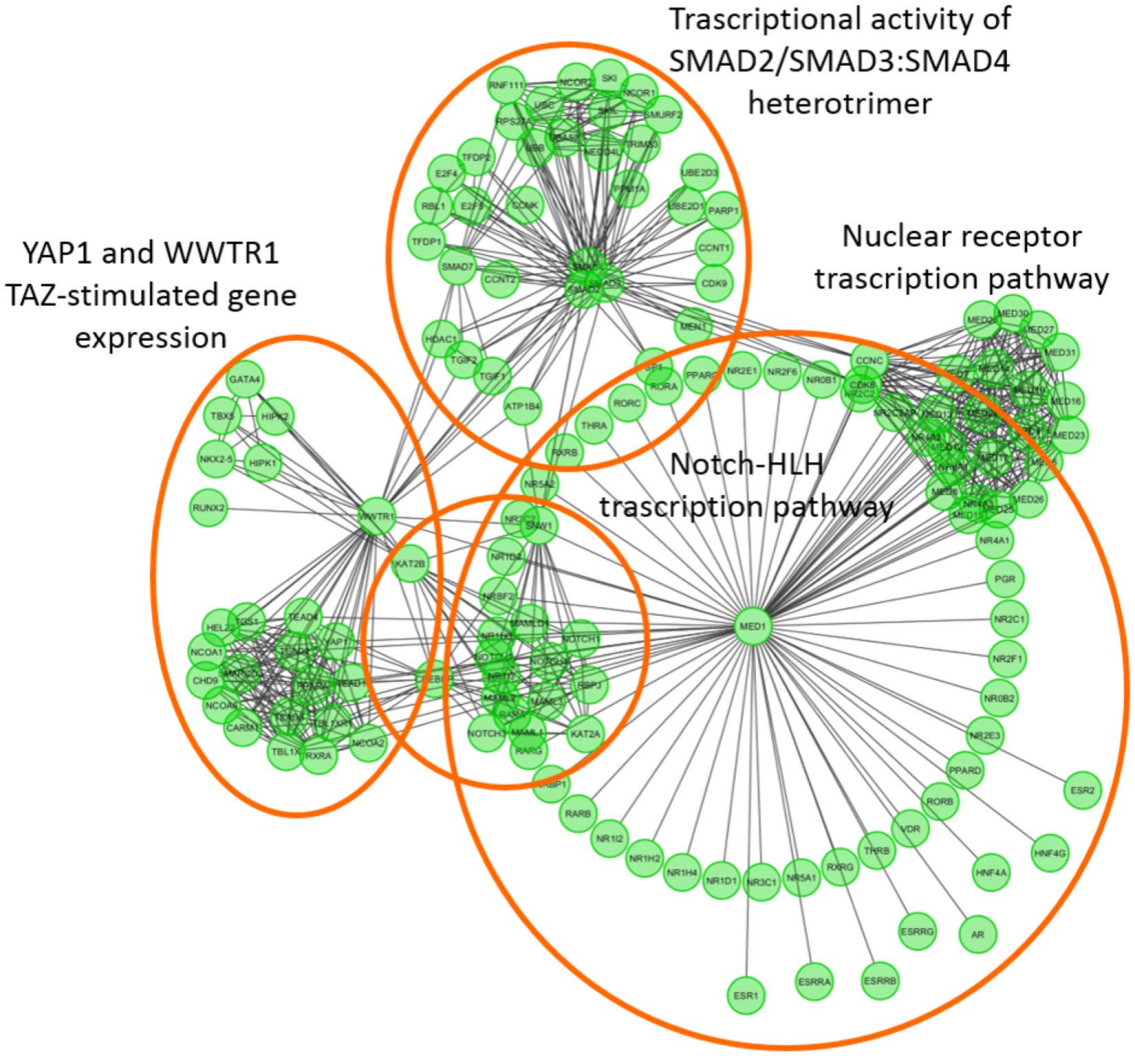
The figure represents the processes at the base of the Generic Transcription pathway (R-HSA-212436). Each process consists of a group of proteins with intra-modular and inter-modular connections. The image has been obtained with ReactomeFVIZ software^14^.

In recent years, the growing amount and quality of –omics data led to the assembly of biological networks, whose ultimate goal is to unveil the underlying cellular processes. In this scenario, Protein-Protein Interactions (PPIs) are among the most important and widely studied networks^1,2^. In PPI networks, a biological system is described in terms of proteins, i.e. the nodes, and their relationships (physical/functional interactions), i.e. the edges. The widespread of PPI networks is justified by their versatility, promoting applications, for example in –omics data integration^3^, protein function discovery^4^, molecular mechanism comprehension^5^, and drug discovery or drug repositioning^6^. The interpretation of PPI networks is therefore a key step to understand the represented system. Given the network sizes, typically involving thousands of elements, it often requires *in-silico* automated methods^7,8^. PPI networks are analyzed through the identification of subnetworks, or modules, showing specific topological and/or functional characteristics^9–13^. A PPI module represents a group of proteins taking part in specific, separable functions such as protein complexes, metabolic pathways or signal transduction systems. A module is identified on the basis of its double role (i) as an isolated entity, being responsible of specific steps of the cellular processes; and (ii) as part of a connection pattern, in which a process influences another one to perform higher-level cellular functions^11^. For example, the Generic Transcription pathway (R-HSA-212436)^14^ achieves its functions through its sub-processes, such as the nuclear Receptor Transcription pathway, the Notch-HLH Transcription pathway, etc. (Fig. 1). In turn, each sub-process can be described as a module made of proteins and other molecules working together to perform a specific step of a bigger pattern.

In network biology and graph theory, it is possible to define *topological* and *functional* modules^15^. The first term refers to a group of nodes having much more connections with the nodes of the group rather than with the ones outside of it. The second term refers to a group of nodes sharing a common biological function. Note that a group of nodes representing a module might possess *both* topological and functional properties. Ideally, the topological and functional modules would coincide; in practice, they constitute two different entities, though typically they largely overlap^9^. As a consequence, both the network topology and the functional information contribute to the overall comprehension of the PPI network biological mechanisms. Topological properties are measured with specific metrics such as modularity, betweenness, degree distribution, density, closeness^10,16^. On the other hand, functional properties are widely described by the three Gene Ontology (GO) categories of Biological Process, Molecular Function, and Cellular Component^17^.

Several graph-based algorithms have been developed to tackle PPI module identification. Most of these approaches infer the modules relying solely on their topological properties. These methods exploit community detection algorithms developed for generic graphs, readjusting them to the context of biological networks^16,18^. Representative methods include Markov Cluster (MCL)^19^, MCODE^20^, CFinder^21^, COACH^22^ and ClusterOne^23^. While the topological approach is sound in network theory, it is sub-optimal in the case of PPI networks, because of their biological nature they present specific limits. For example, the scarce sensitivity of PPI discovery techniques (such as yeast two-hybrid method and tandem affinity purification coupled with mass-spectrometry) leads to the presence of noise, in form of falsely detected edges^24^. Moreover, module identification algorithms mainly focus on the detection of densely connected subgraphs, ignoring functional modules that are often sparsely connected^15,25^, and/or very small, i.e. composed of only two or three proteins^26,27^. Cutting off these modules means to exclude key proteins influencing/driving the inspected biological process. To overcome the issues of noisy edges and small/sparse module detection, some recent algorithms pre-process the network with a-priori knowledge, such as co-expression relations and/or functional associations. In practice, they filter out the low reliability edges, and/or enrich the network with edge weights^28–31^. Despite the integration of a priori information, nonetheless module identification in these algorithms remains strictly topological. A further possibility, so far little explored, is the development of new algorithms relying on other properties of the network and not only on topological ones. In this paper, we describe MTGO (Module detection via Topological information and Gene Ontology knowledge), a novel algorithm we developed to identify modules in PPI networks. It combines information from network topology and knowledge on the biological role of proteins. In order to identify interesting modules, MTGO employs repeated partitions of the network; in this way it reshapes modules on the basis of both the GO annotations and the graph modularity (i.e. a function measuring the topological quality of a partition in a graph). Therefore, the partition is learned through a process of optimization taking into account the network structure as well as its biological nature. Differently from previous approaches based on GO, such as DCAFP^32^ and GMFTP^33^, MTGO provides a unique GO term that best describes the biological nature of each identified module. This supports a better explanation of the results obtained, highlighting the main processes involved in the biological system represented by PPI network models. Because of its unique way of GO exploitation, MTGO differs from state of the art algorithms, where GOs are not directly leading module assembling.

In this paper, we show how MTGO provides a better module identification in different literature-benchmarked networks and target module sets (i.e. ground truth complexes), and in particular we demonstrate that it greatly increases the detection of sparse and small modules. We also show the ability of MTGO to detect functionally significant modules and to find significant GO terms linked to the modules. Finally, we present an example of application to display as MTGO can be used for the analysis of a PPI network and how it can improve the network interpretation.

## Results

We applied MTGO to benchmark PPI scenarios, and compared its results with seven, including also the most recent GO-based, state-of-the-art algorithms. We assess the performances of the considered approaches both from a network-wide perspective, and focusing on the detection of small and sparse modules only. Results are analysed to validate the significance level (i) of the modules found from a functional perspective (with respect to the others GO-based algorithms); and (ii) of the GO terms selected by MTGO to describe the biological mechanisms. Since GO annotations assume a key role in the MTGO algorithm, a section is dedicated to the assessment of the GO contribution to final predictions. Finally, the last section presents an example of MTGO application for the analysis and the interpretation of a Myocardial infarction PPI Network.

### Data collections for nine scenarios

To evaluate the performance of MTGO, four real PPI networks have been selected, including Krogan^34^, Gavin^35^, Collins^36^, and DIP Hsapi^37^ PPI networks. We also assembled a fifth, large network obtained by the integration of all experimental Yeast networks. The first three networks and the integrated network were built using yeast *Saccharomyces Cerevisiae* data, while DIP Hsapi network was built with Human data. Although the three networks of *Saccharomyces Cerevisiae* are in part overlapped, as they come from the same organism, it is important to test all of them because they are obtained with different experimental processes. The presence of false-positive edges and noise in a network is strictly dependent upon the experiment used to detect PPI, thus networks characterized by different noise sources should be used to test the robustness of module identification algorithms. Table 1 shows the main characteristics of each network, including the number of nodes covered by GO terms, used as input for MTGO.

**Table 1:** PPI network characteristics.

	Nodes	GO-covered nodes	Edges
Krogan	2709	2537	7123
Gavin	1856	1778	7669
Collins	1622	1596	9074
Human	2734	2474	4058
Integrated	3232	3020	16948

This functional information has been retrieved downloading the annotation files submitted by GO Consortium members related to *Saccharomyces Cerevisiae* and *Homo Sapiens*. The GO terms used as input for MTGO include all the three categories of Cellular Component, Biological Process and Molecular Function. On the basis of reliability, we retrieved only the GO terms tagged with an Experimental evidence and/or computational analysis evidence Score^17^.

To evaluate the predicted modules with MTGO, gold standard protein complexes have been used as target sets, in particular CYC2008^38^, and the union of MIPS^39^ and SGD^40^, for *Saccharomyces Cerevisiae* PPI networks; and CORUM^41^ for Human PPI network. Protein complexes made of just one protein have been excluded. The curated complexes in CYC2008, MIPS + SGD and CORUM are 408, 509 and 1765, respectively. This led to nine *scenarios*, i.e. eight for *Saccharomyces Cerevisiae* networks (Krogan, Gavin and Collins, and Integrated) against CYC2008 and MIPS + SGD target sets; and one for Human network against CORUM target set.

### Comparison with other approaches

To evaluate the effectiveness of MTGO, results were compared with seven state-of-the-art algorithms. In particular, we compared MTGO with ClusterOne^23^, MCODE^20^, COACH^22^, CFinder^21^, Markov Cluster (MCL)^19^ and DCAFP^32^ and GMFTP^33^. While the first five algorithms are based only on topological properties, DCAFP and GMFTP, similarly to MTGO, exploit functional GO information as well. All the algorithms were run with default parameters, with the exception of the *k* parameter in CFinder, which has been chosen as the best among *k* = 4, 5 or 6 for each run. Note that this range is considered ideal for biological networks, as it is advised in literature^23^. MTGO parameters were set to default for Human network (*minSize* = 2 and *maxSize* = 100); for *Saccharomyces Cerevisiae*, on the other hand, *maxSize* was set to 80, according to the size of the biggest target complex^38^ (for a detailed description of MTGO parameters see Supplementary Materials, Section 1.5).

Although MTGO is able to process both weighted and unweighted networks (a comparison of the two options is provided in the Discussion), since some of the seven chosen algorithms can elaborate just unweighted networks, all the comparisons have been made with unweighted networks (the weights of the networks Krogan, Collins and Gavin have been ignored).

**Figure 2:**
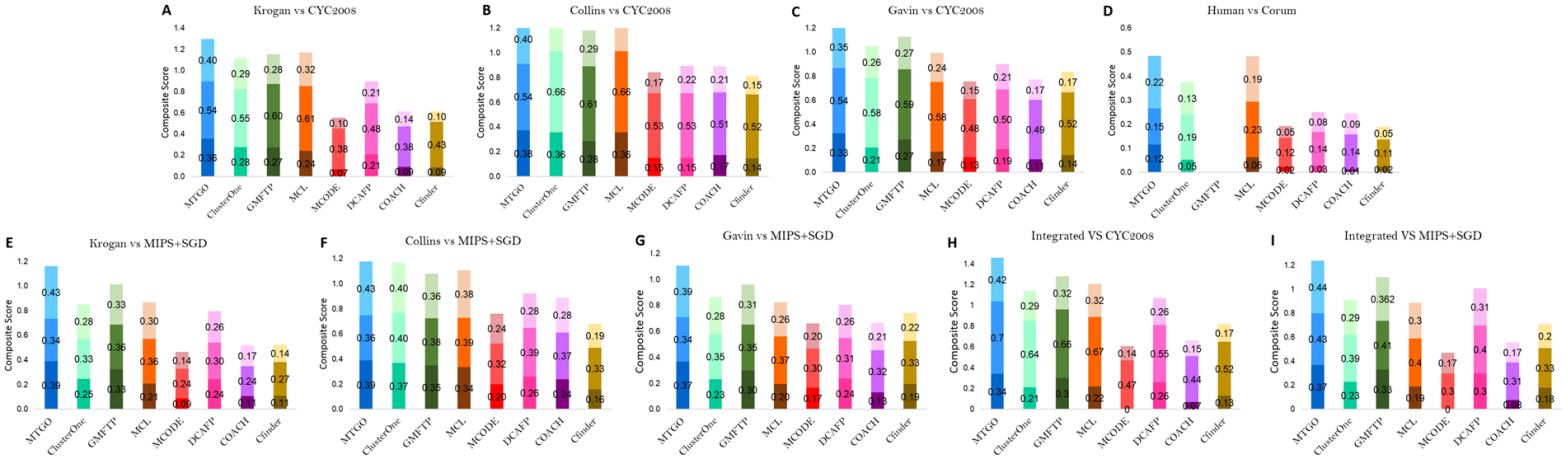
Composite Score of the methods over the different scenarios: MMR (light shade), Accuracy (neutral shade), and Recall (dark shade). GMFTP did not converge on the Human network.

Three independent measures were used to compare predicted complexes with the target sets: *Recall*, *Accuracy*^42^ and *Maximum Matching Ratio* (MMR)^23^ (detailed formulas and further considerations are included in the Supplementary Materials, Section 2). We also measured the *Composite Score*, a comprehensive measure specifically introduced to assess module identification algorithms^23,43^. The Composite Score is calculated as the sum of Recall, Accuracy and MMR. The overall performance of MTGO and its competing algorithms on the nine scenarios is depicted in Fig. 2. These results, along with more detailed measures, including *F-measure*, *Precision*, *Sensitivity*, *N*_*APC*_, |*PC*|, *N*_*ATC*_, |*TC*| and *PPV* are reported in Supplementary Table S1. Note that the performance of GMFTP on the Human network (Fig. 2) is not recorded since the algorithm did not converge after multiple attempts.

MTGO showed the best overall performance in eight out of nine scenarios (best *Composite Score*, *Recall* and *MMR*, see Supplementary Table S1). *Recall* is particularly high, for example in the Human scenario, where *Recall* is doubled compared to the second best algorithm (MTGO 0.12, MCL 0.06; MTGO and MCL unveil 203 vs 111 modules respectively). Note that reaching a high *Recall* is one of the major challenges for module identification algorithms^26^. The worst performance of MTGO is on the Collins vs. CYC2008 scenario, where nonetheless it reaches the third best *Composite Score* (MTGO 1.31 vs ClusterONE 1.42). Interestingly, in the close scenario Collins vs. MIPS + SGD, where protein complexes are different, MTGO shows the best *Composite Score* (MTGO 1.18 vs ClusterONE 1.16).

### Small and Sparse complexes

**Figure 3:**
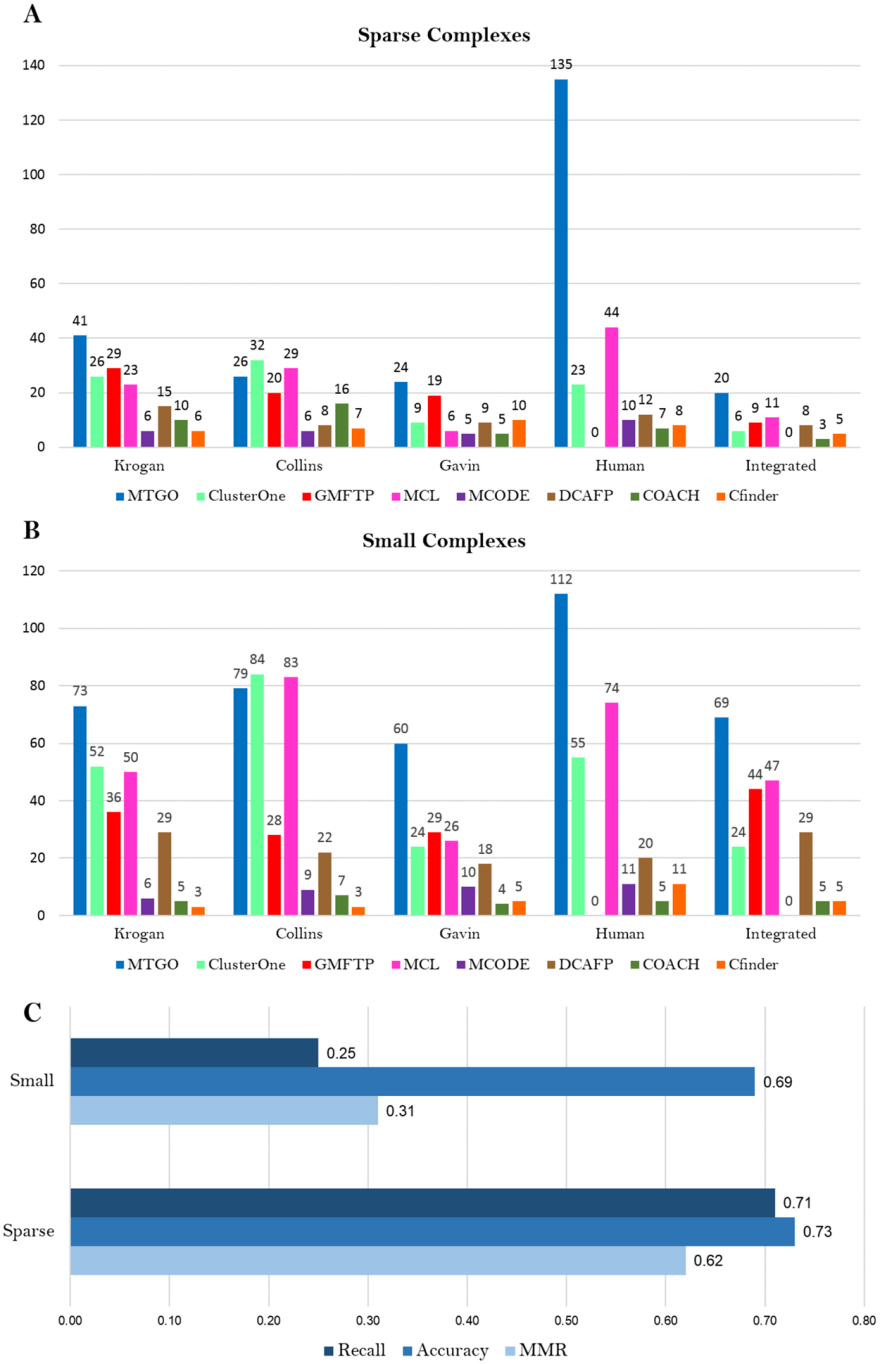
(**A**) Sparse complexes comparison. (**B**) Small complexes comparison. GMFTP did not converge on the Human network. As for Integrated network, MCODE did not predict any complex with Affinity Score^50^ greater than the used threshold 0.5 (Affinity Score formula (S7) and other details are reported in Supplementary Material, Section 2). (**C**) BioGrid Network Small/Sparse complexes detection.

An open problem in module identification algorithms is the detection of small and sparse complexes. While small complexes are defined has having three nodes or less^25^, there is no clear consensus about how to define sparse ones^15,25,26^. We defined five additional scenarios (one per network) to assess both small and sparse module detection. As regards sparse complexes, five different target sets have been created for each network, Krogan, Collins, Gavin, Human and Integrated. As a matter of fact, the same target complex shows different density values according the network considered. Each target set has been created selecting the subset of complexes with density lower than 0.5 with respect to the network considered from the whole target set (CYC2008 for Krogan, Collins, Gavin, Integrated; and CORUM for Human). For example, for the Krogan network the target set of sparse complexes is made of the CYC2008 complex subset showing a density of less than 0.5 with respect to the krogan network. As regards small complexes, two target sets were assembled by considering complexes made of three nodes or less from CYC2008 and CORUM sets. Predicted complexes were compared to target sets using the affinity score (Supplementary Formula S7 in Supplementary Materials, Section 2). Figure 3 shows results for small and sparse complex detection.

Moreover, to test MTGO ability in detecting Small/Sparse complexes in a very large network, the whole BioGrid^44^ network has been processed. The predicted complexes have been compared with two target sets, specific for small and sparse complexes (computed following the same method used for the other five networks, as described above). The predicted complexes have been compared with the two target sets using three independent measures *Maximum Matching Ratio* (MMR)^23^, *Accuracy* and *Recall*^42^ (detailed formulas and further considerations are included in the Supplementary Materials, Section 2).

MTGO outperforms all other algorithms in all scenarios, except in the Collins network. The performances on Human scenarios are remarkably high, especially in detecting sparse modules, MTGO correctly identifies 135 modules, while the second best MCL only 44, less than one third (Fig. 3). Moreover, MTGO can be used to detect Small/Sparse complexes also in very large Networks, as shown by the results obtained for the BioGrid Network (Fig. 3 (C)), where a remarkably high Accuracy has been found (0.69 (Small) and 0.73 (Sparse)).

### GO term analysis

**Figure 4:**
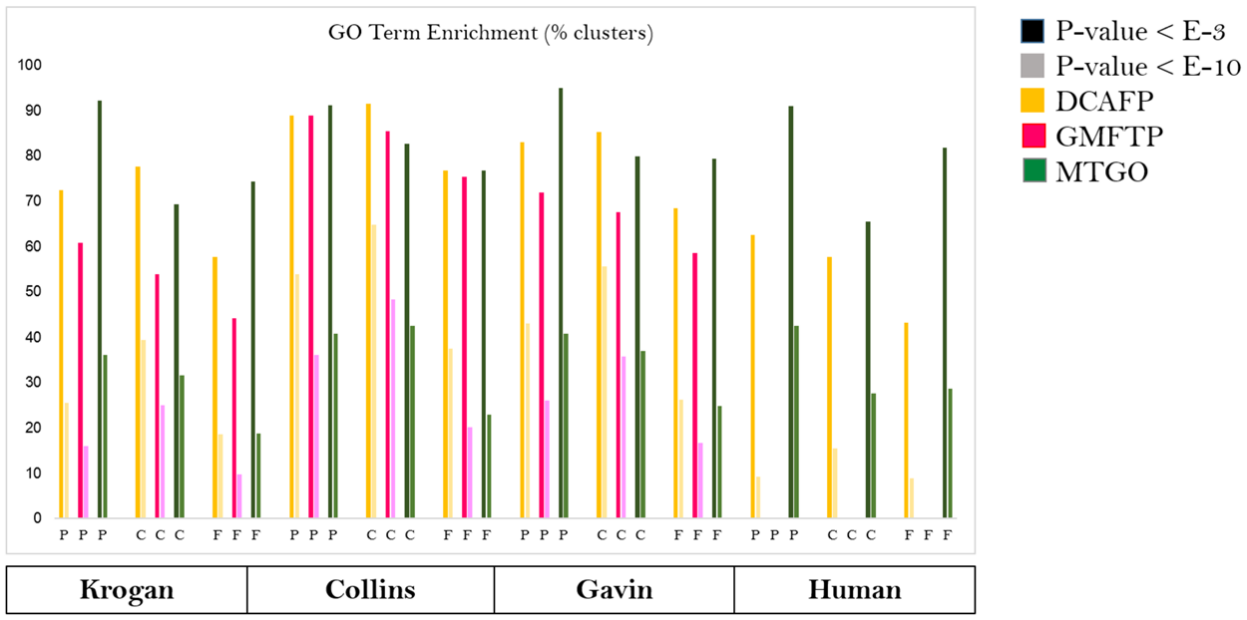
GO term enrichment. P, C and F indicate the three GO classes, respectively Biological Process, Cellular Component and Molecular Function. GMFTP did not converge on the Human network.

In the literature, given a chosen p-value as threshold, a predicted module is defined as functionally significant if at least one GO term is significantly enriched (i.e. associated with a p-value lower than the threshold) in the module proteins^32^. For the protein complexes predicted in each network, we used GOTermFinder^45^ to perform the function enrichment test with 10^−3^ and 10^−10^ p-value thresholds. We compared our results with DCAFP and GMFTP, both GO-based as MTGO. The results are reported in Fig. 4 and in Supplementary Table S2. MTGO labels each module with a specific GO term. To further validate our results, we measured the p-values (Fisher’s exact test) of the GO terms MTGO attributed to each topological module. Table 2 reports the percentage of the MTGO-assigned modules associated to a significant GO term for each analyzed network, considering two different p-value thresholds and Bonferroni correction for multiple testing 10^−3^ and 10^−10^.

**Table 2:** Percentage of significant MTGO-attached GO terms.

	10^−3^	10^−10^
Krogan	96%	49%
Gavin	89%	44%
Collins	81%	39%
Human	94%	59%

### GO contribution to results

**Figure 5:**
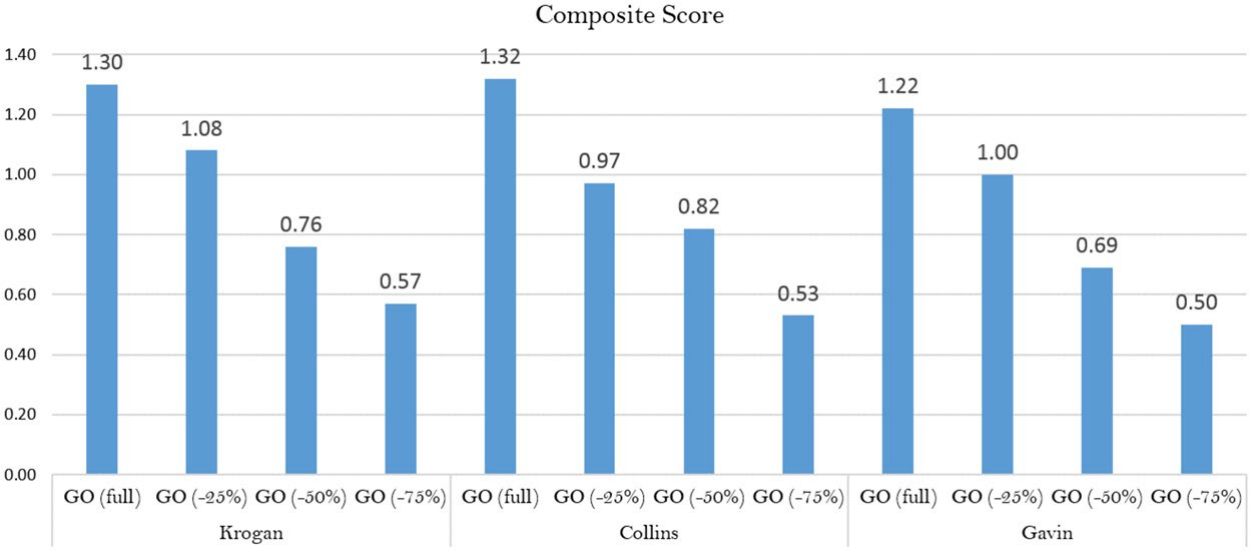
Comparison of MTGO predictions in case of full GO annotations and in presence of perturbed GO annotations (25%, 50% and 75%) in the three networks Krogan, Collins, and Gavin.

We designed a targeted experiment to evaluate the extent the GO contribution to the performance of MTGO. MTGO has been run with a lists of perturbed GO annotations. In particular, to simulate a lower quality GO, we resolved to randomly remove an increasing percentage of proteins from GO terms used by MTGO, with thresholds fixed at 25%, 50% and 75%. For each threshold, we run MTGO over Krogan, Collins, and Gavin networks. We compared the predicted modules with the target set CYC2008^38^, using the Composite Score (Fig. 5). The results show a clear correlation between the percentage of GO terms removed and the decrease performance of MTGO. The highest threshold (75%) corresponds to a Composite Score decrement of 58.6% (mean value respect the three networks), while the smallest threshold (25%) causes a Composite Score average decrease of 20%.

### Myocardial infarction: a case study

**Figure 6:**
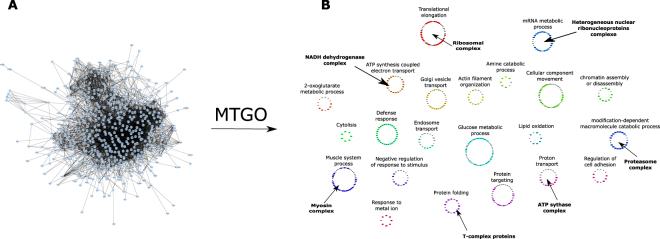
Application of MTGO algorithm to process an experimentally-derived PPI network. (**A**) Myocardial infarction PPI network consisting in 502 nodes and 4316 physical interactions. The network structure derives from Cytoscape following the application of the Organic layout. (**B**) Myocardial infarction PPI network following MTGO algorithm. Circular modules shown in panel (**B**) correspond to topological modules obtained by MTGO (Supplementary Table S3), each one is tagged with the corresponding GO term. Finally, the protein complexes associated with the assigned GO terms are indicated in bold. Node details are explained in Supplementary Figure 6.

To show an application of MTGO on real data, we considered an undirected PPI network obtained by analyzing the proteomics of swine heart tissues affected by myocardial infarction (MI) and treated by human mesenchymal stem cells^46^. The network is made of 502 nodes (differentially expressed proteins) and 4316 edges consisting in physical PPIs (Fig. 6, panel A). Although it may be considered a network of medium size, its structure is too complex to be manually interpreted. We used minSize = 5, maxSize = 30, and a list of 1256 Biological Process GO terms (obtained with Cytoscape plug-in Bingo^47^) related to the network nodes. By tagging modules with GO terms, MTGO successfully outlined well known heart physiology processes (Fig. 6, panel B), including ATP synthesis coupled to electron transport, muscle system process, regulation of cell adhesion or lipid oxidation, and glucose metabolic process, all in agreement with the investigated samples. This structure may be more easily interpreted by biologists and further improve the identification of processes and functions modulated in the considered phenotypes^46^. Moreover, many of these processes are associated also to well defined protein groups, showing the attitude of MTGO to correctly identify molecular complexes (ribosomal complex, heterogeneous nuclear ribonucleoprotein complex, myosin complex, ATP synthase complex, Proteasome complex, T-complex proteins, NADH dehydrogenase complex; see Fig. 6 panel B and Supplementary Table S3) In biologically realistic fashion, MTGO lets functional module overlap, i.e. sharing nodes (proteins). This is achieved via GO terms attribution (Supplementary Figure 7 depicts the network without PPIs, with nodes representing proteins and GO terms connected by belongs to edges).

## Discussion

In this paper we presented MTGO, a novel method to identify functional modules in PPI networks. MTGO theoretical architecture is based on the optimization of both GO term attribution and topology measures. MTGO provides both overlapping and full network coverage, two optimal features for module identification algorithms^15^. In particular, MTGO provides a map of both topological and functional modules. Topological modules ensure full coverage of the network, while functional modules share nodes, *de facto* allowing overlapping. On the other hand, it must be noted that MTGO does not consider topological overlapping (i.e. the modularity function evaluates the likelihood of a partition). MTGO heavily depends on the quality of the associated GO, therefore if this is not well represented; it lacks information; it is biased; or it shows a low *N*_*GO*_ (i.e. number of nodes with at least one GO-associated term), the results are affected negatively. In these cases, the user might consider to use the results optimized for density (see Supplementary Materials, Section 1.6).

Although MTGO is an algorithm designed purposely to use GO annotations, it is also able to work with weighted networks. In fact, the Modularity function, on which it is based, is designed to work both on unweighted and weighted networks^48^. To test the performance of MTGO in both cases, the three networks Krogan, Gavin and Collins have been processed as weighted and unweighted networks. The results show that the use of weights slightly improves the predictions. To evaluate the results, the Composite Score (the sum of *Recall*, *Accuracy* and *Maximum Matching Ratio*) has been computed in both weighted and unweighted cases. In detail, for Krogan network it increases of 4%, for Collins network it is the same in both cases weighted/unweighted and for Gavin it increases of 0.8% (see Supplementary Figure 8 in Supplementary Materials Section 5). Tested on benchmark scenarios, MTGO provides results better than state of the art algorithms in eight scenarios of nine (Fig. 2). By optimizing a trade-off between GO terms and topology, MTGO is extremely accurate in unveiling small and/or sparse functional modules, often missed by other algorithms. Both in the research of sparse and small complexes, MTGO outperforms all other seven algorithms, in four networks out of five. Moreover, MTGO can be used to detect Small/Sparse complexes also in very large Networks, as shown by the high *Accuracy* reached in the BioGrid Network (Fig. 3 (C)).

The high reliability of MTGO-retrieved modules is confirmed by GO term enriched analysis, with associated p-values comparable to or better than other GO-based state of the art algorithms. Overall, by considering the sum of the enriched terms in all the three GO classes (Biological Process, Molecular Function, Cellular Component), MTGO outperforms DCAFP and GMFTP in all the networks but Collins (where DCAFP gets the best performance, consistently with the previously discussed Composite Score results). Nonetheless, MTGO outperforms DCAFP and GMFTP on the biological process related GOs in all the four networks (Supplementary Table S2 and Fig. 4). Furthermore, the superiority of MTGO is clear in the Human network, where MTGO is able to retrieve a particularly high percentage of modules with at least one significant GO term. Compared to DCAFP for p-values of 10^−3^ and 10^−10^ respectively, MTGO retrieves 91% (vs 62%) and 55% (vs 42%) for Biological Process related GO terms; 65% (vs 57%) and 27% (vs 15%) for Cellular Component related GO terms; 81% (vs 43%) and 28% (vs 8%) for Molecular Function related GO terms. Note that GMFTP results are not shown for the Human network as the algorithm failed to provide a viable result after multiple attempts.

MTGO has ability to detect a set of GO terms providing a meaningful biological interpretation of the PPI Network. This is confirmed by the high percentage of modules tagged with significant GO terms. We found the great majority of GO terms (81% to 96% in all four networks) to be significant (<0.001) and about a half (39% to 59%) to be highly significant (10^−10^), both calculated after Bonferroni correction (Table 2).

The output of state-of-art algorithms provides just a set of topological modules without any biological interpretation, thus further analyses are needed to investigate the biological meaning of the results. MTGO, thanks to its unique characteristics (it provides both a network partition and a set of GO terms describing it), allows to couple in a single step two different types of network analysis, topological and functional.

Clearly, the performance of MTGO are affected by the completeness of GO annotations, however MTGO is designed to work even if the annotations are incomplete (in Table 1 shows that the number of GO-covered nodes is always smaller than the node number). To evaluate the GO annotation contribution on the MTGO final prediction a targeted experiment has been designed. As expected, the MTGO performance gets worse when the input GO term list is reduced by removing proteins. However, when the entity of the reduction is little (25%) the Composite Score gets worse of a little percentage (20%), ensuring a good result anyway. Although the incompleteness of the GO annotations could be a disadvantage of the method, the original use of the GO and the combination with topological network properties give to MTGO a clear advantage in module searching, as demonstrated by the MTGO superiority reached in eight different scenarios against seven different algorithms.

MTGO time complexity analysis is reported in the Supplementary Materials Section 6.

As a future direction, we aim to exploit the functional/topological module identification of MTGO to define the *disease modules*^9^. This application is particularly interesting for Protein Co-expression Networks, a technique to build protein functional networks exploiting directly the protein expression profiles coming from organic sample analysis. Protein co-expression networks are a graph where edges represent protein relations in the specific physiological/pathological context analyzed^10^. MTGO has the ability to select a subset of GO terms describing a protein network, i.e. each GO term selected is biologically linked to a protein subset represented in the network in form of nodes sharing an high number of edges. For this reason, the application of MTGO on a Protein Co-expression Network allows to exploit at most its ability, because the edges are directly inferred from the biological system investigated. In this way, the comparison of MTGO functional and topological sets in case (disease) vs control (healthy) networks would pinpoint the GO term difference and network rewiring characterizing the analyzed disease. In other words, explicitly addressing the disrupted/altered cellular functions.

In summary, MTGO is viable tool to speed up PPI network analysis by automatically discovery of functional modules.

## Methods

### Input and output

**Figure 7:**
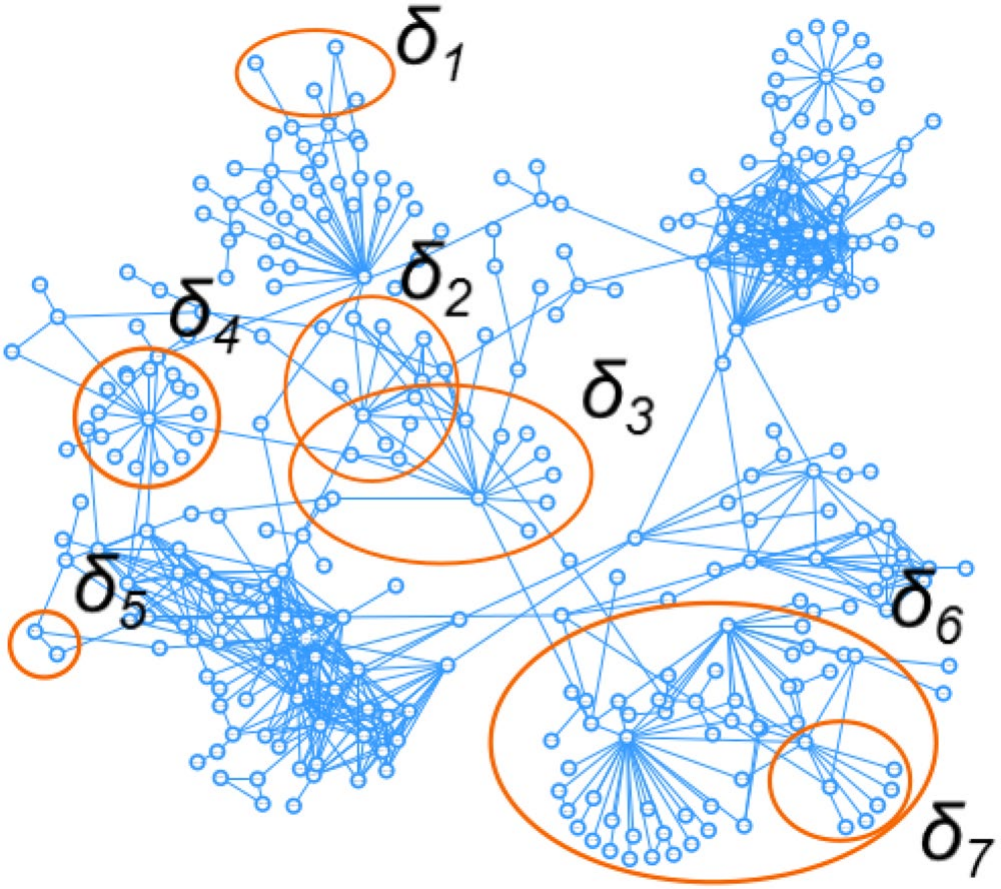
Example of *δ* elements represented in a network, they may share more nodes or be included into a bigger category.

A *PPI* network can be represented as *G* = (*V*, *E*), where *V* and *E* are the nodes and edges of the network, respectively. *V* is the set of proteins and it is defined as *V* = {*v*_1_, *v*_2_, *v*_3_, …, *v*_*N*_}, with *N* is the total number of proteins/nodes. *E* represents the set of the relationships between network nodes and it is defined as $E=\{{e}_{{\rm{i}},{\rm{j}}}\},(i,j)\in [1,N]$. Therefore, *G* carries the *PPI* topological properties. In order to integrate biological function information in the PPI Network, we can assign GO terms to the network nodes. Given a user-provided list of GO terms (e.g. the entire GO or a sub-list, see MTGO User Manual for further details), MTGO computes the set *T* = (*L*, Δ), where the *p*−*th* element is *t*_*p*_ = (*l*_*p*_, *δ*_*p*_), *l*_*p*_ is the ontology term, while *δ*_*p*_ is the *l*_*p*_-associated set of network proteins. Examples of the network *δ*_*p*_ elements and their structure are shown in Fig. 7. Note that if a GO term of the input list is not associated with any network protein, MTGO automatically filters it out.

*I* = (*G*, *T*) is the input of the system. The goal of MTGO is to process *G* to find groups of nodes sharing both the topological (*V*, *E*), and the functional (*T*) properties. The result of MTGO is the final output *R*^*F*^ = (*C*^*F*^, Φ^*F*^), where *C*^*F*^ is the set of the topological modules, Φ^*F*^ is the set of functional modules, and *H* is the total number of both topological module set and functional module set, i.e. |*C*| = |Φ| = *H*. The relation between the elements of *C* and Φ is 1:1. MTGO iteratively computes *C* and Φ, and the pair *R*^*F*^ = (*C*^*F*^, Φ^*F*^) is selected as final output. Note that modules are generally called *clusters* in literature. Since MTGO considers two different kinds of modules, here for clarity and simplicity we will not use the term cluster, but *topological* and *functional* modules. The model *R* is a global representation of the system in terms of modules, each one with a topological (*C*^*F*^) and a functional (Φ^*F*^) representation. The set of the topological modules *C* is a partition of the network, defined as *C* = {*c*_1_, ..., *c*_h_, ..., *c*_H_} such that:

1 $${c}_{1}\cap {c}_{2}\ldots \cap {c}_{{\rm{h}}}\ldots \cap {c}_{{\rm{H}}}\equiv \rlap{/}{0};\quad \quad {c}_{1}\cup {c}_{2}\ldots \cup {c}_{{\rm{h}}}\ldots \cup {c}_{{\rm{H}}}\equiv V;$$

Note that by definition, each node of a partition *C* is uniquely assigned to a single topological module. The set Φ = {*φ*_1_, …, *φ*_h_, …, *φ*_H_}, on the other hand, describes the functional modules involved in the network. Φ is defined as follows:

2 $${\phi }_{1}\cap {\phi }_{2}\ldots \cap {\phi }_{{\rm{h}}}\ldots \cap {\phi }_{{\rm{H}}}\ne \rlap{/}{0};\quad \quad {\phi }_{1}\cup {\phi }_{2}\ldots \cup {\phi }_{{\rm{h}}}\ldots \cup {\phi }_{{\rm{H}}}\subseteq V$$

where Φ ⊂ *T*, i.e. Φ is the subset of *T* selected by MTGO to describe the biological functions linked to the partition *C* of the PPI network.

*Full coverage* and *overlapping* are considered the ideal features of module identification algorithms^15^. MTGO grants both with its dual complementary output *C* and Φ, respectively. In particular, the *C* topological modules represent a network partition, thus granting full coverage by definition. On the other hand, the Φ functional modules *overlap*, allowing the assignment of a node to two or more modules. This feature is particularly important since it reflects the behavior of biological systems, where a protein may be involved in multiple functions.

#### MTGO algorithm

**Figure 8:**
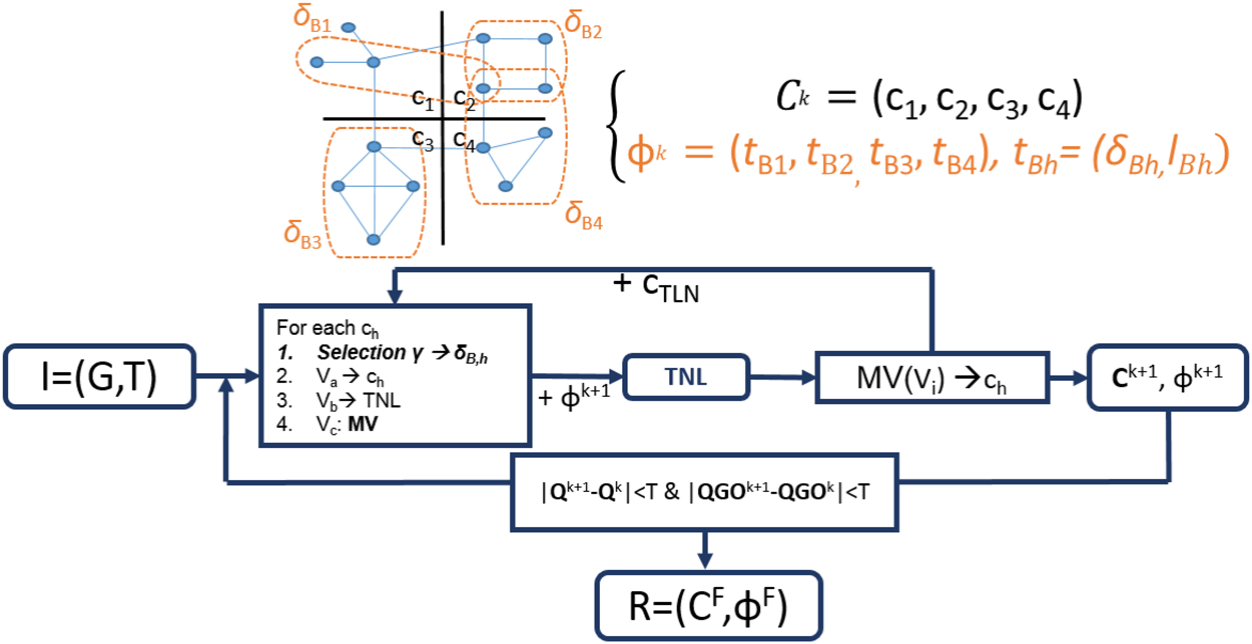
Workflow of MTGO. Iteratively, MTGO associates the functional module *δ*_*Bh*_ optimizing *γ* for each topological module *c*_*h*_. Nodes of module *c*_*h*_ are redistributed according to the sets *V*_*a*_, *V*_*b*_ and *V*_*c*_. Hard-to-assign nodes are at first moved to the Temporary Node List (TNL). The TNL is emptied either moving its nodes to existing *c*_*h*_ s or to the newly created topological module *c*_*TLN*_. At each iteration *k*, the output is a pair (*C*^*k*+1^, Φ^*k*+1^). MTGO checks threshold *T* for steady state. If reached, the pair *C*^*F*^, Φ^*F*^ is the final output.

In the following, we provide a description of MTGO. Given the input *I* = (*G*, *T*), MTGO performs its tasks in three main phases: (i) initialization; (ii) iteration; and (iii) check for convergence. MTGO whole process is summed up in Fig. 8.

### Initialization

**Figure 9:**
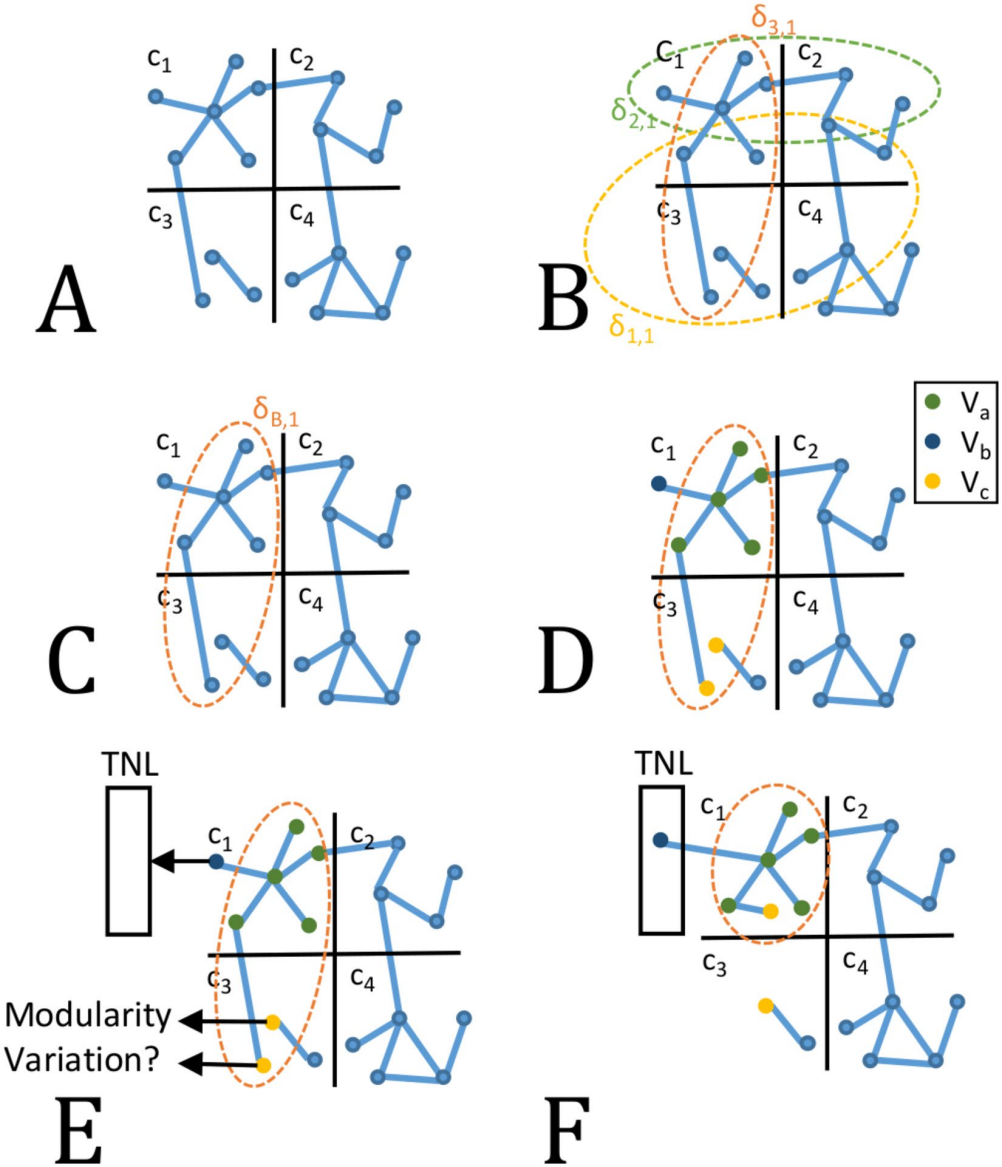
Iteration Phase of MTGO. Nodes are assigned to topological modules *c*_*h*_ (Panel A). Functional modules *δ* fit topological modules differently. For example, *δ*_1,1_, *δ*_2,1_, and *δ*_3,1_, overlap differently with *c*_1_. The best functional module is *δ*_3,1_, since it minimizes the number of nodes out of the intersection between *c*_1_ and itself. It is then selected as *δ*_*B*,1_ (Panels B and C). Once *δ*_*B*,1_ is selected, the nodes of *δ*_*B*,1_∪*c*_1_ are grouped into three sets: *V*_*a*_, *V*_*b*_, and *V*_*c*_ (Panel D). *V*_*a*_ are the nodes shared by *δ*_*B*,1_ and *c*_1_; *V*_*b*_ are the nodes belonging to *c*_1_ but not to *δ*_*B*,1_; *V*_*c*_ are the nodes belonging to *δ*_*B*,1_ but not to *c*_1_. *V*_*a*_ nodes stay in *c*_1_; *V*_*b*_ nodes are moved to the TNL; *V*_*c*_ nodes either remain in their topological module *c*_3_, or are moved to *c*_1_, according to the Modularity Variation function. Here, one *V*_*c*_ node is embedded in *c*_1_, while the other stay within its original topological module *c*_3_.

In the initialization phase, *V* is used to create a random partition *C*^0^ (Fig. 9, Panel A), in which the number of topological modules is $\propto \sqrt{N}$. *T* is created from a GO term list provided by the user, according to the set *V*. Two user-defined parameters, *minSize* and *maxSize*, set the minimum and maximum size of *T* modules respectively, i.e. the minimum and maximum number of nodes in a *δ*_*p*_.

### Iteration

MTGO follows an iterative process. At each iteration, a pair (*C*, Φ) is computed: *C* by re-assigning the nodes of the previous partition, and Φ by selecting elements from *T* that best describe *C*. Each partition *C* is made of topological modules *c*_*h*_ with *h* representing the index of the single topological module and 1 ≤ *h* ≤ *H*; (the total number of functional modules *H* varies at each iteration). Ideally, MTGO aims to assign nodes such that topological modules coincide with functional modules. In detail, the iteration phase is performed with two main sub-processes.

#### Step 1

Topological modules are randomly processed at each iteration. Each *c*_*h*_ is processed as described in Fig. 9. Firstly, *δ*_*B*,*h*_ is selected from the group of all the *δ*s associated to *c*_*h*_, i.e. the *δ*s containing at least one node of *c*_*h*_ (Fig. 9, Panels B and C). *δ*_*B*,*h*_ is the element minimizing the *Selection* function *γ*, i.e. the one minimizing the number of not included nodes in *c*_*h*_ ∩ *δ*_*h*_. (*Selection* function *γ* is described in detail in Supplementary Materials Section 1.2 and Supplementary Figure 2). The assignment of *δ*_*B*,*h*_ to *c*_*h*_ defines three node sets *V*_*a*_, *V*_*b*_ and *V*_*c*_. *V*_*a*_ is the set of nodes shared by *δ*_*B*,*h*_ and *c*_*h*_; *V*_*b*_ is the set of nodes belonging to *c*_*h*_ but not to *δ*_*B*,*h*_; *V*_*c*_ is the set of nodes belonging to *δ*_*B*,*h*_ but not to *c*_*h*_. Note that *V*_*c*_ nodes belong to other topological modules of the partition (Fig. 9, Panel D). From here, nodes in *c*_*h*_ are re-assigned as follows:

*V*_*a*_ nodes remain in the topological module *c*_*h*_.

*V*_*b*_ nodes are moved to the *Temporary Node List* (TNL). The TNL is a temporary repository of nodes discarded from their original topological modules, and waiting to be re-assigned (Fig. 9, Panel E).

**Figure 10:**
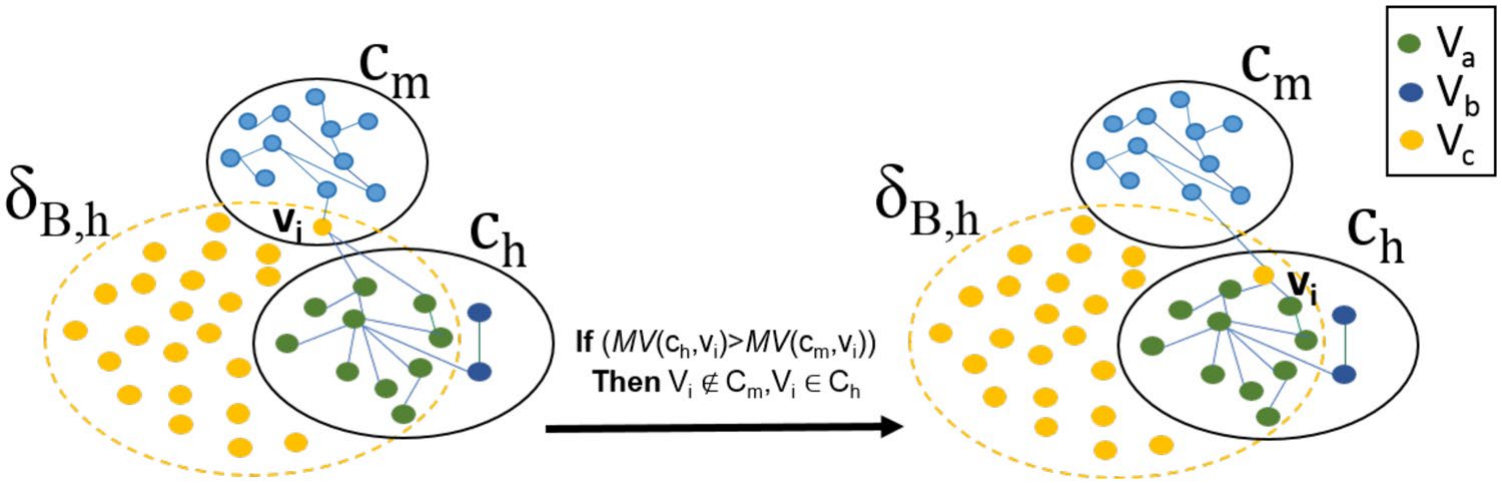
*V*_*c*_ node repositioning. The node *v*_*i*_, belonging to *δ*_B,h_ and *c*_*m*_ moves to *c*_*h*_ topological module if *MV*(*c*_*h*_, *v*_*i*_) *MV*(*c*_*m*_, *v*_*i*_).

*V*_*c*_ nodes can either stay in their original topological module *c*_*m*_ (*m* ≠ *h*) or be assigned to *c*_*h*_, as they are biologically related to it, since they share *δ*_*B*,*h*_. A node *v*_*i*_ ∈ *V*_*c*_ is moved to *c*_*h*_ if it increases the global Modularity^16^ (see formula (3)), according to a Modularity Variation (*MV*) function, and in particular if *MV*(*c*_*h*_, *v*_*i*_) *MV*(*c*_*m*_, *v*_*i*_) (details in the Supplementary Materials Section 1.3, and Fig. 10).

#### Step 2

**Figure 11:**
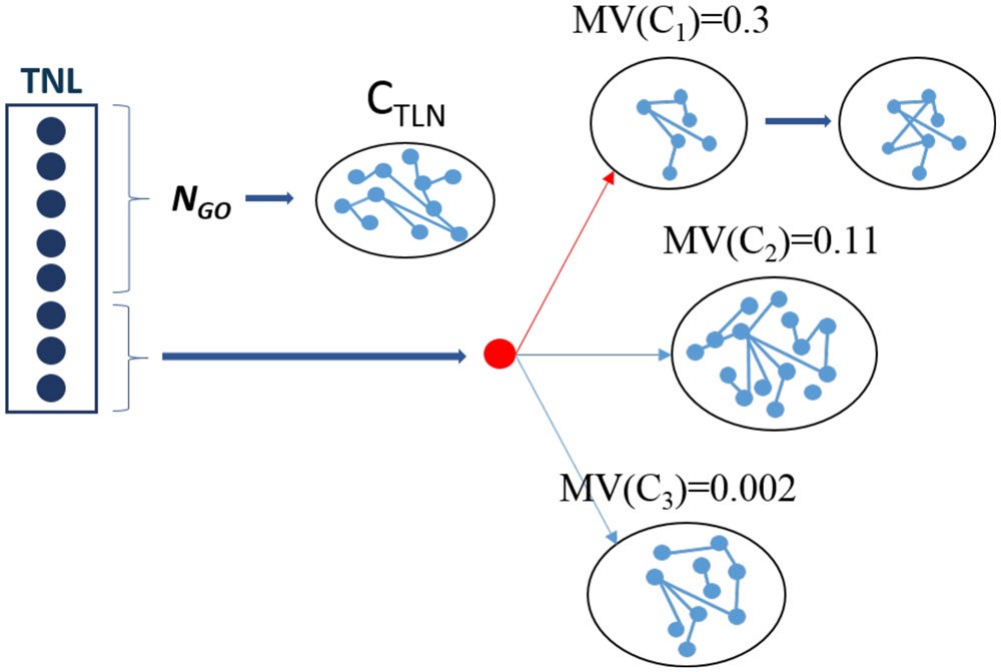
Step 2, the TNL is emptied. The nodes with at least one GO term (*N*_*GO*_), the first TNL five nodes, are grouped to generate a new topological module *c*_*TLN*_. Nodes without any GO term, the last three TNL nodes, are assigned to the topological module that maximizes the *MV*. In this example, the red node is assigned to the topological module *c*_1_, showing the max value of *MV*.

In this step the TNL nodes are re-assigned. All the TNL nodes with at least one associated *δ*, *N*_*GO*_, are used to create a new topological module *c*_*TLN*_. It is worthwhile to note that *N*_*GO*_ is a subset of the total nodes present in the PPI Network, some nodes may not be covered by any GO term. While, each node *v*_*i*_ without any associated *δ* is assigned to the existing topological module optimizing the *MV* function (Fig. 11). *c*_*TLN*_ is integrated into the network through the repetition of Step 1.

At the end of the Iteration phase, MTGO outputs the selected functional modules *δ*_*B*, *h*_s, along with their linked *l*_*B*,*h*_s, grouped into Φ, and the newly computed topological modules *c*_*h*_s, grouped into *C*.

Note that a detailed version of the MTGO Iteration phase is provided in the Supplementary Materials Section 1.

### Check for convergence

Two different functions are used to check if the convergence is reached: modularity (*Q*)^49^ and Quality GO (*QGO*). *Q* evaluates the global quality of the partition *C*, while *QGO* evaluates the agreement between *C* and Φ. Ideally, *C* and Φ should overlap. The *Q* formula is:

3 $$Q({C}^{k})=\sum _{1 < h < {H}_{k}}\frac{{e}_{h}^{k}}{|E|}-{(\frac{{d}_{h}^{k}}{2\ast |E|})}^{2}$$

Here, the index *k* indicates the *k*-*th* iteration of the algorithm. Thus, *C*^*k*^ is the *k*-*th* partition; *H*^*k*^ is the number of topological modules; ${e}_{h}^{k}$ is the total number of edges in the *h-th* topological module; ${d}_{h}^{k}$ is the sum of the node degrees of the *h*-*th* topological module. *Q* values range from −1 to 1, with positive values if there are more links within topological modules than expected at random, and negative otherwise. *Modularity Q* is the most popular function to evaluate the graph partitions^16^. While, the *QGO* formula is:

4 $$QGO({C}^{k})=\frac{{\sum }_{1 < h < {H}_{k}}|{\delta }_{B,h}^{k}\cap {c}_{h}^{k}|}{{N}_{GO}}$$

Here ${\delta }_{B,h}^{k}$ is the functional module minimizing the *Selection γ* function for the topological module ${c}_{h}^{k}$ (see *Iteration* Section, Step 1); and *N*_*GO*_ is the total number of nodes with at least one *δ*_*p*_ assigned. *QGO* evaluates the degree of overlapping between *C*^*k*^ and Φ^*k*^.

Set a threshold *T*, the steady state is reached when |*Q*^*k*+1^ − *Q*^*k*^| < *T and* |*QGO*^*k*^ − *QGO*^*k*−1^| < *T*. The solution *R* = (*C*^*F*^, Φ^*F*^) is taken as the one with maximum value of *QGO*. The set *C*^*F*^ is the partition maximizing *QGO*, while the set Φ^*F*^ is the set of all pairs ${t}_{B,h}^{F}=({\delta }_{B,h}^{F},{l}_{B,h}^{F})$ assigned for each ${c}_{h}^{F}$ topological module. Note that in our experiments, we set *T* = 10 − 4.

### Data availability

The datasets generated during and/or analysed during the current study are available from the corresponding author on reasonable request.

## Electronic supplementary material

Supplementary Table 1

Supplementary Table 2

Supplementary Table 3

Supplementary Table 4

Supplementary Materials
