# Supplementary material for: MTGO: PPI Network Analysis Via Topological and Functional Module Identification
Source: Sci Rep. 2018 Apr 3;8:5499. doi: 10.1038/s41598-018-23672-0 (PMC5882952; doi:10.1038/s41598-018-23672-0)
Supplement: Supplementary file 5 — Supplementary Materials [file 41598_2018_23672_MOESM5_ESM.pdf]

Supplementary Materials

MTGO: PPI network analysis via topological and functional module identification

Danila Vella, Simone Marini, Francesca Vitali, Dario Di Silvestre, Giancarlo Mauri, and Riccardo Bellazzi.

Contents

1 Details of the MTGO algorithm **2**

1.1 MTGO local metrics 2

1.2 Selection function 2

1.3 MV function 2

1.4 Details of the Iteration phase 3

1.5 Parameters 6

1.6 Density optimization result 8

2 Evaluation Formulas **8**

3 Q and QGO trends **9**

4 Case Study Figures **10**

5 Weighted VS Un-weighted Figure **12**

6 Computational complexity analysis **12**

1 Details of the MTGO algorithm

1.1 MTGO local metrics

The MTGO algorithm is based on two main functions: *Modularity Variantion* MV, as regards topological aspects; and *Selection* γ, as regards biological aspects. These two functions are applied *locally* on each single module during the building process. In contrast, their *global* counterparts *Modularity* (topological) and *QGO* (biological) are applied to the whole network (see Materials and Methods in the main text). γ is used to assign to a single topological module c_h a pair $t_B = (l_B, \delta_B)$, where l_B is the GO term best describing the biological meaning of c_h, and δ_B is (i) the list of network nodes associated to l_B, and (ii) the functional module linked to c_h. As consequence, nodes are added/removed to let the topological module fit its assigned functional module. The main constraint of module modification is represented by the MV function. MV allows the topological nature of the module to be preserved. In fact, it is possible to add a node only if, by adding it, the module topology is ameliorated.

1.2 Selection function

We define the γ function as

$$\gamma(\delta^k_{p,h}) = \frac{|\delta^k_{p,h}| - |\delta^k_{p,h} \cap c^k_h|}{|\delta^k_{p,h}| - 1} + \frac{|c^k_h| - |\delta^k_{p,h} \cap c^k_h|}{|c^k_h| - 1} \tag{S1}$$

where c^k_h is the *h-th* topological module of the iteration k; $\delta^k_{p,h}$ is the *p-th* element of Δ^k_h; and Δ^k_h is a subset of the set Δ for the nodes of c^k_h.

The aim of *Selection* γ function is to choose a GO term (represented by $\delta^k_{B,h}$) as model to drive the building process of a topological module.

$$\delta^k_{B,h} = \underset{\delta^k_{p,h} \in \Delta^k_h}{\text{argmin}} \gamma(\delta^k_{p,h}) \tag{S2}$$

The GO term assigned to a topological module should assure a good fitting, thus proving *both* a high overlapping and high specificity. In general, if a GO term has high degree of overlapping, it is very likely that the GO term is little specific for the topological module and vice-versa. In fact, the choice of a GO term involving all the topological module nodes could lead to select a low specificity GO term. A low specificity GO term contains many nodes not belonging to the topological module (i.e. included in other topological modules in the network). This problem is depicted in Supplementary Figure 1. *Selection* γ is designed, therefore, to find a trade off between overlapping and specificity. Supplementary Figure 2 shows the behavior of the *Selection* function.

1.3 MV function

We define the MV function as

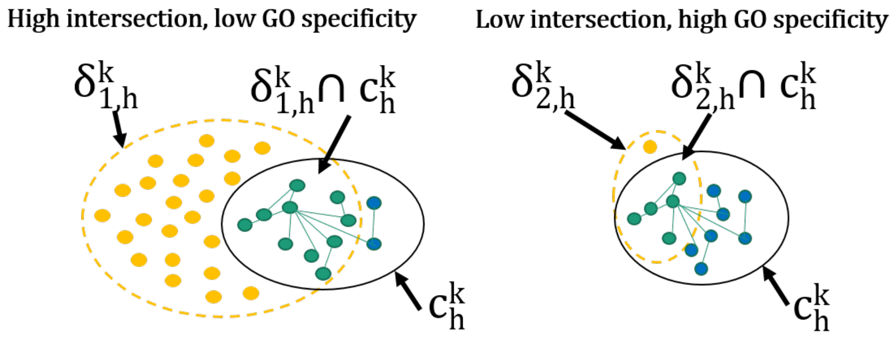

Supplementary Figure 1: Two cases of fitting of δs to a topological module c_h^k. In the first case, $\delta_{1,h}^k$ has a good overlap with the topological module, involving almost all the nodes (green nodes), but it tags many other nodes (yellow nodes) outside of the topological module as well. Thus, $\delta_{1,h}^k$ is not very specific to this topological module. In the second case, $\delta_{2,h}^k$ is very specific for the topological module, in effect almost all $\delta_{2,h}^k$ nodes are included in it, but it has a low overlap (green nodes).

$$MV(c_h^k, v_\mathrm{i}) = q(c_h^k + v_\mathrm{i}) - q(c_h^k - v_\mathrm{i}) \tag{S3}$$

Where q (S4) represents the contribute of a single topological module to global function Modularity Q (S5); $c_h^k + v_\mathrm{i}$ indicates the topological module including v_i, while $c_h^k - v_\mathrm{i}$ indicates the topological module without v_i. Defining q as the modularity contribute of c_h^k, MV calculates the variation of q due to adding the node v_i.

$$q(c_h^k) = \frac{e_h^k}{|E|} - \left(\frac{d_h^k}{2 * |E|}\right)^2 \tag{S4}$$

$$Q(C^k) = \sum_{1<h<H^k} \frac{e_h^k}{|E|} - \left(\frac{d_h^k}{2 * |E|}\right)^2 \tag{S5}$$

C^k is the k-th partition and H^k is its total topological module number; e_h^k is the total number of edges in the h-th topological module; d_h^k is the sum of the node degrees of the h-th topological module. *Modularity* Q is the sum of $q(c_h^k)$s over the c_h^ks of a partition C^k.

1.4 Details of the Iteration phase

MTGO follows an iterative process. At the $k-th$ iteration, a pair (C^k, Φ^k) is computed: C^k by re-assigning the nodes of the previous partition C^{k-1}, and Φ^k

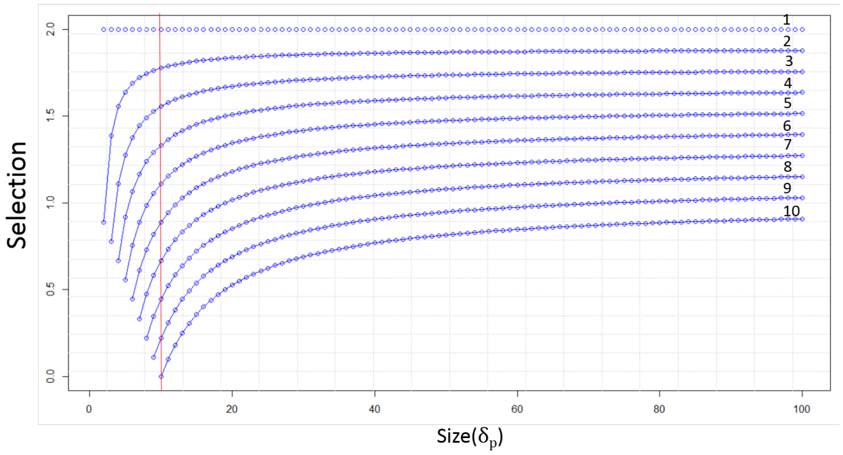

Supplementary Figure 2: The *Selection* function operates a trade-off to associate a δ_p to a topological module, in particular mediating the δ_p nodes internal to the topological module vs. the external ones (Supplementary Figure 1). This graphic shows the behavior of the *Selection* function when the size of the topological module is 10 nodes, and the size of the δ_ps ranges from 1 to 100. Each curve shows the function value for different values of intersection between topological module and δ_p, i.e. how many nodes are internal to module. The intersection values are showed in the right. The *Selection* value decreases according to both the δ_p size shrinking, and to the intersection value increasing.

by selecting elements from T that best describe C^k. Each partition is made of topological modules c_h^k with h representing the index of the single topological module and $1 \leq h \leq H^k$; (The total number of functional modules H^k varies at each iteration.) Ideally, MTGO aims to assign nodes such that topological modules coincide with functional modules. In detail, the iteration phase is performed with two main sub-processes.

Step 1.

Topological modules are randomly processed at each iteration k. Each topological module with $size < minSize$ (see main test Section 1.5 for a parameter description) is discarded and its nodes are added to the Temporary Node List (TNL). The rationale behind TNL is to use it as a temporary repository for discarded nodes. Discarding small topological modules is the way of MTGO to decrease H, i.e. to decrease the number of modules between two consecutive iterations. For each remaining topological module c_h^k, the following four steps are executed:

1.1 Δ_h^k is the subset of Δ containing the elements involving c_h^k nodes; Each $\delta_{p,h}^k$, $p-th$ element of Δ_h^k is evaluated by the *Selection* γ function (S1).

The functional module minimizing *Selection* γ (the *best* functional module $\delta_{B,h}^k$) is assigned to c_h^k.

The attribution of $\delta_{B,h}^k$ to c_h^k defines three node sets V_a, V_B and V_c, as follows:

$$V_a = \delta_{B,h}^k \cap c_h^k; V_B = c_h^k - V_a; V_c = \delta_{B,h}^k - V_a \tag{S6}$$

V_a are the nodes shared by $\delta_{B,h}^k$ and c_h^k; V_B are the nodes belonging to c_h^k but not to $\delta_{B,h}^k$; V_c are the nodes belonging to $\delta_{B,h}^k$ but not to c_h^k. Note that V_c nodes belong to other topological modules of the partition. At the end of Step 1, each c_h^k has its associated $\delta_{B,h}^k$, generating V_a, V_B and V_c.

1.2 Set V_a remains in topological module c_h^k.

1.3 Set V_B is moved to the TNL. V_B nodes are excluded from the $\delta_{B,h}^k$, and therefore they are not significantly related to the biological meaning assigned to c_h^k.

1.4 A single node $v_i \in V_c$ belongs to either another topological module of the current partition C^k, or the TNL (e.g. it has been assigned to the TNL by processing a previous c_m^k and $\delta_{B,m}^k$ pair). v_i is moved to the topological module c_h^k either if (i) it belongs to the TNL; or (ii) if $VM(c_h^k, v_i) > VM(c_m^k, v_i)$ (S3), where c_m^k is the other topological module of C^k containing it. In other words, v_i is added to c_h^k if the topological quality is increased by adding it. Figure 5 in the main text graphically describes this process.

When these four steps are executed for all the topological modules of C^k, MTGO performs another size check. Topological modules with $size < minSize$ are discarded, and their nodes added to the TNL. At this point, all nodes have been assigned either to the topological modules, or to the TNL. Finally, we need to empty the TNL.

Step 2.

In this step the nodes of the TNL are re-assigned to the topological modules:

2.1 All the TNL nodes with at least an associated δ_p, N_{GO} set, are used to create a new topological module c_{TLN} (Figure 6 in the main text).

2.2 Each node v_i without any associated δ_p is assigned to the existing topological module maximizing its $MV(c_h^k, v_i)$ (S3) (Figure 6 in the main text).

2.3 c_{TLN} is the garbage topological module made of the rejected nodes. To integrate it in the partition, *Step 1* is repeated and a second TNL is created. This procedure is the way MTGO introduces new topological modules and increases H in two consecutive iterations. Φ^{k+1} set is obtained here, by grouping all $\delta_{B,h}^k$s and their associated $l_{B,h}^k$s.

2.4 The second TNL is emptied assigning each node v_i to the topological module maximizing its $MV(c_h^k, v_i)$, regardless of having at least an associated δ_p or not.

Now all the nodes of TNL have been assigned to topological modules (full coverage). The obtained topological modules are grouped in the new partition C^{k+1}. While C^{k+1} is computed as described above, Φ^{k+1} is simply the set of all $\delta_{B,h}^k$s, along with their associated $l_{B,h}^k$s.

Detailed pseudocode of the Iteration phase, along with the whole MTGO algorithm are provided in the Supplementary Figure 3.

1.5 Parameters

The parameter *minSize* ranges from 2 to 15 and is used to limit the minimum number of nodes in modules. The parameter *maxSize* ranges from 30 to 300 and is used to limit the maximum number of nodes in modules. Tuning these parameters is useful to adjust the final output in accordance to the needs of a specific study. For example, if the user is interested in identifying a specific process or small protein complexes, *minSize* and *maxSize* should be set to small values (e.g. 2 and 5). In order to identify the general/high level biological processes involved in the network, on the other hand, the user should set *minSize* and *maxSize* to high values (e.g. 10 and 200). Supplementary Figure 4 shows an example of how different results can be obtained from the same network by changing the parameters.

Phase 1. Initialization

- Read inputs: PPI Network *G=(V,E)*, GO list
- Build initial random partition C_0 ($H_0 \sim \sqrt{N}$)
- Build the set T, according to *minSize, maxSize* and *V*

Phase 2. Iteration

$C^k = C^{k-1}$

```
Step 1. For each c_h^k
            If size(c_h^k) > minSize
                δ_{B,h}^k = argmin Selection(δ_p, c_h^k)
                If δ_{B,h}^k ≠ Ø:
                    For each v_i ∈ δ_{B,h}^k & v_i ∉ c_h^k
                        If v_i ∈ TNL: v_i → c_h^k
                        Else If MV(c_h^k, v_i) > MV(c_m^k, v_i) : v_i → c_h^k
                    For each v_i ∈ c_h^k & v_i ∉ δ_{B,h}^k
                        v_i → TNL
                Else c_h^k → TNL
            Else c_h^k → TNL
        For each c_h^k
            If size(c_h^k) < minSize: c_h^k → TNL
Step 2. If Size (N_GO) > minSize:
            N_GO → c_TNL
            ∀ v_i ∈ N_WGO: v_i → c_h^k = argmax MV(c_h^k)
        Else ∀ v_i ∈ TNL: v_i → c_h^k = argmax MV(c_h^k)
        Repeat Step 1 (Compute Φ^{k+1})
        ∀ v_i ∈ second TNL : v_i → c_h^k = argmax MV(c_h^k)
```

Phase 3. Check for steady state

```
If (|Q^{k+1}-Q^k|<T and |QGO^{k+1}-QGO^k|<T)
    C^F = argmax QGO(C^k), Φ^F = (δ_{B,1}^F , δ_{B,2}^F ,… δ_{B,H_F}^F )
Else → Phase 2. Iteration
```

Supplementary Figure 3: MTGO pseudocode

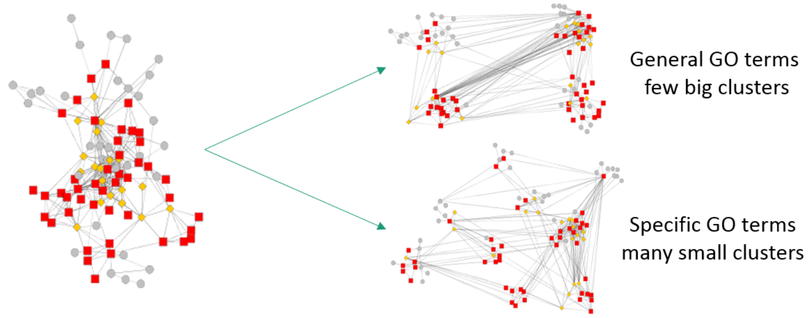

Supplementary Figure 4: Different values of parameters *MaxSize* and *MinSize* lead to different results.

1.6 Density optimization result

MTGO provides also an alternative output R_D based on density optimization, to be used in case of poorly GO-enriched networks (see MTGO User Manual). For example, when the GO term list is very poor and the node percentage covered by the GO terms are under 50%. The solution $R_D = (C^D, \Phi^D)$ is taken as the one with maximum value of mean density over topological modules. For this solution, the set Φ^D is scarce in term of biological quality, while the set C^D preserves its quality in term of topological properties.

2 Evaluation Formulas

We evaluated MTGO and other algorithms according to *Recall*, *Accuracy* [1] and *Maximum Matching Ratio* (MMR). *Recall* is the fraction of true complexes matched by at least one predicted complex over the affinity score (AS) [2], defined as

$$AS(TC, PC) = \frac{|TC \cap PC|^2}{|TC| * |PC|} \tag{S7}$$

where TC stands for Target Complex (module), and PC Predicted Complex (module). For this work, we set the AS threshold to 0.5. Recall is calculated as

$$Recall = \frac{N_{\text{APC}}}{N_{\text{TC}}} \tag{S8}$$

where N_{APC} is the set of PC with $AS > 0.5$. *Accuracy* is defined as

$$Accuracy = \sqrt{Sensitivity * PPV} \tag{S9}$$

Where *Sensitivity* and *Positive Predictive Value* (PPV) are:

$$Sensitivity = \frac{\sum_i \max_j |TC \cap PC|}{\sum_i |TC|} \tag{S10}$$

$$PPV = \frac{\sum_j \max_i |TC \cap PC|}{\sum_j \sum_i |TC \cap PC|} \tag{S11}$$

where i indicates the i-th Target Complex (TC) and j the j-th Predicted Complex (PC) [2].

MMR has been introduced as a specific measure for module identification algorithm [3]. It is based on the maximal one-to-one mapping between PC and TC. It was proposed to overcome an *Accuracy*-related issue in the specific case of module identification algorithms, i.e. the misleading role of *Positive Predictive Value* if some proteins in a TC are present in either more than one PC or in none.

3 Q and QGO trends

Supplementary Figure 5 shows the trend of the functions Q and QGO. These two functions evaluate the global topological and functional properties of each partition. Each red line in Supplementary Figure 5 depicts the value of Q computed with the fast greedy modularity optimization algorithm [4]. The red line can be considered the reference value of the maximum reachable modularity for each network. In the initial iterations the modularity shows a fast increment and it almost reaches its maximum value, while QGO shows a slower, steady increment. After reaching its peak, the modularity decreases, allowing a re-arrangement of the partition in order to improve the GO quality of the topological modules (as supported by the slowly increment of global QGO). However, reaching the last iteration, when QGO reaches its maximum value, Q remains positive (i.e. GO quality increases at the expenses of the best modularity, but without cripple the topological properties of the partition). MTGO provides also an alternative model R_D based on density optimization, to be used in case of poorly GO-enriched networks (see MTGO User Manual and Section 1.6 in the main text).

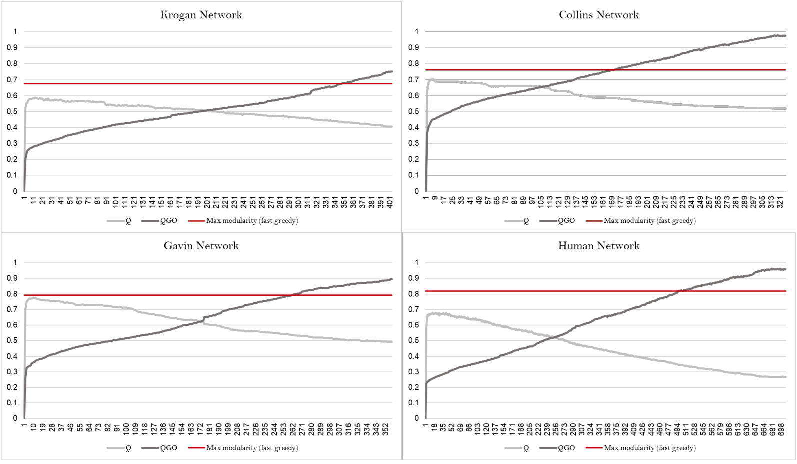

Supplementary Figure 5: Q and QGO trends. Here we show the values of Q and QGO functions for the four networks, computed at each iteration.

4 Case Study Figures

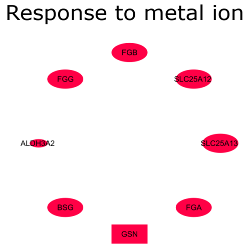

Supplementary Figure 6: Detail of the network represented in Figure 11 in the main text. Small nodes represent proteins excluded from the GO term assigned to the topological module. Big nodes (both circles and rectangles) indicate proteins associate with the same GO term assigned to topological module, while rectangles indicate nodes assigned to more than one functional modules.

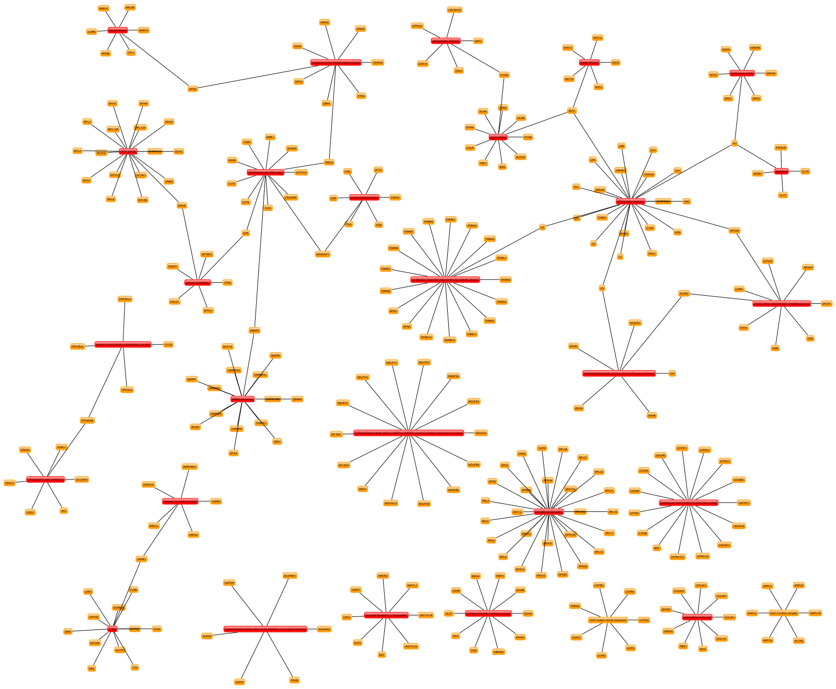

Supplementary Figure 7: Functional modules identified by MTGO for the myocardial infarction PPI network. Here, links indicate protein membership to one or more functional modules, identified by the GO terms. PPI edges are not represented in this figure.

5 Weighted VS Un-weighted Figure

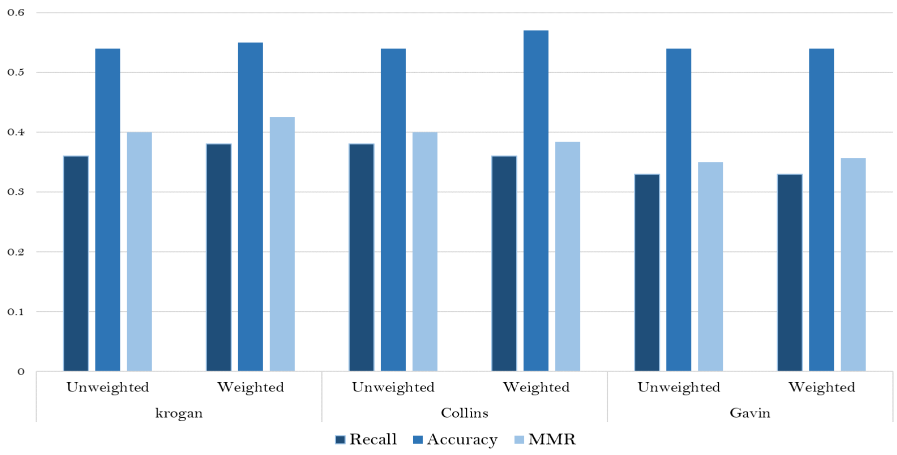

Supplementary Figure 8: MTGO performance comparison with weighted and unweighted networks: Krogan, Collins and Gavin. The two results have been compared using *Recall*, *Accuracy* and *Maximum Matching Ratio*

6 Computational complexity analysis

Table 1 shows the time needed to build networks with different number of nodes and edges on a $Intel-Core^{TM}$ i3-4170 (CPU 3.7GHz) PC with 4GB of RAM. The main aim of MTGO is to evaluate a specific biological context (represented by a PPI Network model) by identifying the main biological functions (represented by GO terms) acting at the base of the system. For this reason, it has been designed to analyze small/middle size PPI networks, which represent a specific tissue or biological condition, as the Myocardial Infarction Network. MTGO scales well with the increase of edges, but not with the increase of the number of nodes. MTGO is able to provide very good results also in big networks, as we showed in the Results section, but with a consistent increase of computation time. In case, the user can take the partial results provided by MTGO each 30 iterations, without waiting for the algorithm to plateau (see User Manual on link https://gitlab.com/d1vella/MTGO).

Computational complexity has been evaluated taking into to the number of nodes (n), the number of iterations (k), the number of nodes in a module (g) (for simplicity, we suppose all the modules contain the same number of nodes), and the number of GO terms (j). Simplifying, the method can be seen as the iteration of two main steps $k[Step1 + Step2]$ (these phases are described in Method Section). The two main functions of the algorithm have complexity

Table 1: MTGO run time

	Nodes	Edges	Time (sec)
Test 1 Network	78	545	1
Test 2 Network	446	24567	120
Myocardial Network	502	4316	30
Gavin Network	1856	7669	10237
Krogan Network	2709	7123	54000

$ModularityVariation \rightarrow \mathcal{O}(ng)$ (S3) and $Selection \rightarrow \mathcal{O}(j)$ (S1). In $Step1$ the two main processes involve firstly (i) the computation of the $Selection$ for each module (the number of modules is $\frac{n}{g}$) and secondly (ii) the computation of $ModularityVariation$ for each module. Therefore, the time complexity of the $Step1$ is $\mathcal{O}(n^2)$. The $Step2$ consists in the computation of the $ModularityVariation$ for each node in the Temporary Node List (TNL) (in the worst case the TNL contains all the network nodes) and for each module, therefore the complexity of the $Step2$ is $\mathcal{O}(n^3)$. This implies that each of the k iterations has time complexity $\mathcal{O}(n^3)$.

References

[1] J. Ji, A. Zhang, C. Liu, X. Quan, Z. Liu, Survey: Functional module detection from protein-protein interaction networks, IEEE Transactions on Knowledge and Data Engineering 26 (2) (2014) 261–277.

[2] X. Li, M. Wu, C.-K. Kwoh, S.-K. Ng, Computational approaches for detecting protein complexes from protein interaction networks: a survey, BMC genomics 11 (1) (2010) S3.

[3] T. Nepusz, H. Yu, A. Paccanaro, Detecting overlapping protein complexes in protein-protein interaction networks, Nature methods 9 (5) (2012) 471–472.

[4] A. Clauset, M. E. Newman, C. Moore, Finding community structure in very large networks, Physical review E 70 (6) (2004) 066111.
